# The In Vitro Antifungal Activity of Sudanese Medicinal Plants against *Madurella mycetomatis*, the Eumycetoma Major Causative Agent

**DOI:** 10.1371/journal.pntd.0003488

**Published:** 2015-03-13

**Authors:** Hassabelrasoul Elfadil, Ahmed Fahal, Wendy Kloezen, Elhadi M. Ahmed, Wendy van de Sande

**Affiliations:** 1 Division of Pharmaceutical Microbiology, Department of Pharmaceutics, Faculty of Pharmacy, University of Gezira, Gezira, Sudan; 2 The Mycetoma Research Centre, University of Khartoum, Khartoum, Sudan; 3 Erasmus MC, Department of Medical Microbiology and Infectious Diseases, Rotterdam, The Netherlands; 4 Department of Pharmacognosy, Faculty of Pharmacy, University of Gezira, Gezira, Sudan; University of Tennessee, UNITED STATES

## Abstract

Eumycetoma is a debilitating chronic inflammatory fungal infection that exists worldwide but it is endemic in many tropical and subtropical regions. The major causative organism is the fungus *Madurella mycetomatis*. The current treatment of eumycetoma is suboptimal and characterized by low cure rate and high recurrence rates. Hence, an alternative therapy is needed to address this. Here we determined the antifungal activity of seven Sudanese medicinal plant species against *Madurella mycetomatis*. Of these, only three species; *Boswellia papyrifera*, *Acacia nubica* and *Nigella sativa*, showed some antifungal activity against *M. mycetomatis* and were further studied. Crude methanol, hexane and defatted methanol extracts of these species were tested for their antifungal activity. *B. papyrifera* had the highest antifungal activity (MIC50 of 1 ug/ml) and it was further fractionated. The crude methanol and the soluble ethyl acetate fractions of *B. papyrifera* showed some antifungal activity. The Gas-Liquid-Chromatography hybrid Mass-Spectrophotometer analysis of these two fractions showed the existence of beta-amyrin, beta-amyrone, beta-Sitosterol and stigmatriene. Stigmatriene had the best antifungal activity, compared to other three phytoconstituents, with an MIC-50 of 32 μg/ml. Although the antifungal activity of the identified phytoconstituents was only limited, the antifungal activity of the complete extracts is more promising, indicating synergism. Furthermore these plant extracts are also known to have anti-inflammatory activity and can stimulate wound-healing; characteristics which might also be of great value in the development of novel therapeutic drugs for this chronic inflammatory disease. Therefore further exploration of these plant species in the treatment of mycetoma is encouraging.

## Author Summary

This study was an attempt to discover new safe therapeutic modalities of natural origin for the treatment eumycetoma. The methanolic extracts of seven selected local plants were screened for their antifungal activity. All seven plant extracts were able to inhibit *Madurella mycetomatis* growth at a concentration of 50 μg/ml or less. Three plant species out of the seven managed to inhibit the growth of *Madurella mycetomatis* at a concentration as low as 781 μg/ml; these were: *Boswellia papyrifera*, *Acacia nubica* and *Nigella sativa*. Further analysis of *B*. *Papyrifera*, using Gas-Liquid-Chromatography hybrid Mass-Spectrophotometer (GC-MS); showed the occurrence of four phytoconstituents, triterpenoid in nature and these were Beta-amyrin, Beta-amyrone, Beta-Sitosterol and Stigmatriene. Stigmatriene was the best to show anti-mycetoma activity at an MIC equal to 32 μg/ml. This MIC is much higher than the MIC of the whole extract (8 μg/ml), indicating that other compounds or a combination of compounds has a better ability to inhibit *M*. *mycetomatis* growth. These results are promising and further detailed studies are highly recommended.

## Introduction

Eumycetoma is a neglected tropical chronic subcutaneous inflammatory disease commonly caused by *Madurella mycetomatis*. It is characterised by a painless subcutaneous mass, multiple discharging sinuses and discharge that usually contains grains [1, 2]. The infection usually progresses to involve the skin, deep structures and ultimately the bone leading to massive deformities, distortions and disabilities [3, 4, 5].

Eumycetoma is reported worldwide but is endemic in tropical and subtropical regions, in what is known as the mycetoma belt and stretches between 15° South and 30° North of the equator [6]. Young active adults are mostly affected [7]. Mycetoma has several adverse effects on patients, communities and health authorities [8, 9].

The available eumycetoma treatment is a combination of massive surgical excisions and long term antifungal therapy [10, 11]. The therapeutic response to this regimen is suboptimal, characterized by low cure and high recurrence rates [12]. Currently ketoconazole and itraconazole are the antifungal drugs of choice. In Sudan, ketoconazole has been the drug of choice. Unfortunately, this drug can cause serious side effects such as hepatic toxicity, and is therefore in 2013 suspended for use by the US Food and Drug Administration and the European Medicines Agency. Furthermore, the drug appeared also expensive for people living in endemic areas, costing as much as a third of their monthly income [13,14]. Officially ketoconazole has now been replaced by itraconazole. Itraconazole is less toxic but unfortunately also appears to be less effective in mycetoma treatment. It creates a tough fibrous tissue around the mycetoma lesions which facilitate their surgical excision [14]. It is interesting to note in these lesions the organisms are commonly present and viable in culture which indicates that, these antifungal drugs are simply localizing the infection and not eradicating it [14]. This perhaps is quite astonishing as many *in vitro* studies showed that *M*. *mycetomatis* is inhibited in growth at low concentrations of these therapeutic agents [15, 16, 17, 18]. But this was explained by host factors and/ or factors from the causative agent such as the grain melanin particularly DHN-melanin which proved to be responsible for this observation [19, 20].

Therefore there is a need for a better and more effective therapy for eumycetoma. An unexplored resource of novel therapeutic agents is the locally growing herbs which are often used in traditional medicine. A recently study conducted at the Mycetoma Research Centre, Sudan showed that, 42.4% of the studied population used herbal medicine for the treatment of eumycetoma at some stage of their illness with a complication rate of 29.3% [21].

Although such a large portion of the mycetoma patients use herbal medicine, there is relatively few information on the antifungal activity of these herbs. We therefore set out this study, to determine the antifungal activity of seven Sudanese plant species used in these local herbal treatments against *M*. *mycetomatis* in the hope to develop an alternative novel treatment for mycetoma in the future.

## Materials and Methods

### Plant species used

In this study seven Sudanese plants species were tested for antifungal activity against 13 independent clinical *M*. *mycetomatis* isolates. The parts tested were gum resin of *Boswellia papyrifera* of the family *Burseraceae*, root bark of *Acacia nubica* of the *Mimosaceae* family, seeds and fruits of *Nigella sativa* of the family *Renunculaceae*, flower buds of *Eugenia caryophyllus* of the family *Myrtaceae*, rhizome of *Zingiber officinalis* of the family *Zingiberaceae*, stem peel of *Cinnamon verum* of the family *Lauraceae* and fruits of *Piper nigrum* of the family *Piperaceae*.

### Preparations of the plant extracts

The plant extracts were prepared from dried coarsely powdered samples macerated separately in conical flasks with methanol over seven days at room temperature. Following filtration, crude methanolic extracts were dried under vacuum using a rotatory evaporator at 60°C. The extracts were dissolved separately in 70% alcohol and subjected to liquid-liquid partitioning using hexane to obtain three products: a crude methanol extract, a hexane fraction and a defatted methanol fraction as described previously. [22, 23, 24]

### Identification of potential antifungal components in the crude extracts of *Boswellia papyrifera*

To determine which components in these extracts were responsible for the antifungal activity of *Boswellia papyrifera*, methanol extracts were fractionated using a separating funnel successively with hexane and ethyl acetate to produce four fractions: an exhausted methanol fraction, a hexane fraction, a crude methanol extract and an ethyl acetate fraction. The fraction in which the antifungal activity was detected was further characterized by Gas liquid Chromatography hybrid Mass spectrophotometry (GC-MS) analysis.

For this extracts were dissolved in ethyl acetate, and one μl injections were made in split mode at injection temperature 280°C. Initial temperature was 80°C, then increased at 10°C/min; compounds were identified by comparison of their mass spectra with those from the NIST’98 mass spectral database. The identified triterpenoid phytoconstituents were Beta-amyrin, Beta-amyrone, Beta-sitosterol and Stigmatriene. To test the antifungal activities of these phytoconstituents, the pure compounds were purchased by Sigma.

### Determining the *in vitro* activities of the different extracts and compounds against *M*. *mycetomatis*

The broth microdilution method was used to determine the crude methanol extract, hexane fraction, defatted methanol fraction, crude methanol fraction, exhausted soluble methanol fraction, soluble hexane fraction, soluble ethyl acetate fraction, Beta-amyrin, Beta-amyrone, Beta-sitosterol and Stigmatriene antifungal activity against clinical *M*. *mycetomatis* isolates

Each of the *M*. *mycetomatis* isolates was identified to the species level by sequencing the ITS region [15–18]. The procedure started with the culturing of *M*. *mycetomatis* for 10 days at 37° in RPMI-1640 medium supplemented with L-glutamine (0.3g/L) and 6 ml MOPS of 0.165 M. The resulting mycelia were harvested by 5 minutes centrifugation at 2158 g and washed with normal saline. The mycelia were homogenized by sonicating for 20 seconds at 28 micron, using a sonicator (Mesonix-CEMODEL3510E-MT-Mexico S/Nqpko40517794). The final inoculum was prepared by adjusting the homogenized fungal suspension to obtain an optical transmission of 70% at 660 nm. Of this suspension 100 microliters were transferred into a microtitration plate. To each well 2 μl of a two-fold dilution of each extract in DMSO was added. The microtitration plate was incubated for 7 days at 37°C before the endpoints were read. The MIC was determined to be the first well in which no growth was visible. Biological assays were compared to negative, positive; inoculum and solvent controls. As a comparator agent, ketoconazole was applied in all plates. All assays were performed in triplicate. To facilitate the endpoint reading, XTT was added after incubation as described elsewhere [22].

## Results

### The extracts from *Boswellia papyrifera* appear to have the most potent antifungal activity against *M*. *mycetomatis*

In order to determine if seven of the locally used medicinal plants have any antifungal activity against *M*. *mycetomatis*, crude methanol extracts of *E*. *caryophillus*, *C*. *verum*, *P*. *nigrum*, *Z*. *officinalis*, *A*. *nubica*, *N*. *sativa and B*. *papyrifera* were prepared (Table 1). All the studied methanol extracts were able to inhibit *M*. *mycetomatis* growth at a concentration of 50 μg/ml. Three of these extracts had a more potent antifungal activity and these were *A*. *nubica*, *N*. *sativa* and *B*. *papyrifera*. They inhibited *M*. *mycetomatis* growth at a concentration as low as 1 μg/ml (Table 1)]. Hence, these three plant species were selected for further study. Crude methanol extracts, hexane extracts and the defatted methanol extracts were prepared from these plant species and their antifungal activities were determined for the clinical *M*. *mycetomatis* isolates. Low MICs were found for all three extracts, with MIC-50s ranging from 1 μg/ml to 4 μg/ml (Table 1).

**Table 1:** In vitro antifungal activities of several extracts of seven locally plant species against 13 *M*. *mycetomatis* isolates.

	MIC 50^1^ (range) in μg/ml						
Plant species	Crude methanol extract	Hexane extract	Defatted methanol extract	Crude methanol fraction	Exhausted soluble methanol fraction	Souble hexane fraction	Soluble ethyl acetate fraction
*Eugenia caryophillus*	50 (ND^2^)	ND (ND)	ND (ND)	ND (ND)	ND (ND)	ND (ND)	ND (ND)
*Cinnamum verum*	25 (ND)	ND (ND)	ND (ND)	ND (ND)	ND (ND)	ND (ND)	ND (ND)
*Piper nigrum*	25 (ND)	ND (ND)	ND (ND)	ND (ND)	ND (ND)	ND (ND)	ND (ND)
*Zingiber officinalis*	12.5 (ND)	ND (ND)	ND (ND)	ND (ND)	ND (ND)	ND (ND)	ND (ND)
*Acacia nubica*	1 (0.5–128)	2 (0.25–128)	4 (0.5–128)	ND (ND)	ND (ND)	ND (ND)	ND (ND)
*Nigella sativa*	1 (0.5–128)	2 (0.25–128)	4 (0.25–128)	ND (ND)	ND (ND)	ND (ND)	ND (ND)
*Boswellia papyrifera*	1 (0.5–128)	1 (0.25–128)	2 (0.25–128)	8 (8)	128 (128)	128 (128)	8 (8)
Ketoconazole	0.125 (0.03–0.25)						

^1^Minimal inhibitory concentrations (MIC) at which at least 50% of the *M*. *mycetomatis* isolates was inhibited (MIC50).

^2^ND: not done.

The most potent antifungal activity was documented in the crude methanol extracts of *B*. *papyrifera* (Table 1). The crude methanol fraction of *B*. *papyrifera* was further fractionated into four different fractions: a crude methanol fraction, an exhausted soluble methanol fraction, a soluble hexane fraction and a soluble ethyl acetate fraction to determine in which of these fractions the antifungal activity was present. The *M*. *mycetomatis* growth was inhibited only by the crude methanol fraction and the soluble ethyl acetate fraction, (Table 1).

To determine the exact nature of the antifungal compounds, GC-MS analyses were performed on the crude methanol extract and the soluble ethyl acetate fractions. It appeared that in both extracts high amounts of tetracyclic and pentacyclic triterpenoid compounds were present. These compounds were identified as: Beta-amyrin, Beta-amyrone, Beta-sitosterol and Stigmatriene based on the comparison of their retention times and masses with those from the NIST’98 mass spectral database (Table 2). Strikingly, only Beta-amyrone and Stigmatriene were present in both extracts in relatively high concentrations.

**Table 2:** GC-MS analysis presenting triterpenes identified by GC/MS in *Boswellia papyrifera* extracts.

Fraction	Retention time	% area	Base peak	Molecular weigth	Identification of compound
Crude methanol	20.614	18.24	218	424	beta-amyrone
	20.757	0.48	392	410	Stigmatriene
	22.09	20.17	218	426	beta-amyrin
soluble ethyl acetate	20.614	12.46	218	424	beta-amyrone
	20.757	72.41	392	410	Stigmatriene
	21.752	2.53	129	486	beta-sitosterol

Since in the active fractions of the *B*. *papyrifera* extracts; high concentrations of Beta-amyrin, Beta amyrone, Beta-sitosterol and Stigmatriene were found, it was our plan to determine if any of these phytoconstituents can exhibit antifungal activity against *M*. *mycetomatis*. Findings revealed that, Beta-amyrin, beta-amyrone and beta-sitosterol only had limited activity against *M*. *mycetomatis* (Table 3). Only four isolates were inhibited by lower concentrations of beta-amyrin and beta-amyrone and only two for beta-sitosterol. The other isolates were not inhibited by these compounds at the highest concentration tested. For stigmatriene most isolates were inhibited at lower concentrations and only four isolates were not inhibited at all. But even for stigmatriene the overall MICs obtained were relatively high, compared to the whole extract.

**Table 3:** In vitro antifungal activities identified of triterpenoidal compounds from the *B. papyrifera* extracts against 12 *M*. *mycetomatis* isolates.

Compound	MIC50 (range) in μg/ml
beta-amyrin	>256 (0.5->256)
beta-amyrone	>128 (0.25 - >128)
beta-sitosterol	>128 (0.125 - >128)
stigmatriene	32 (0.125->128)

## Discussion

In many centers extensive eumycetoma lesions are still treated with a combination of massive surgical excisions (or amputation) and prolonged antifungal therapy [10,11]. These treatment modalities are based on personal clinical experience and a few case reports rather than controlled clinical trials [10]. The treatment outcome is appalling and dreadful characterized by low cure rate, high recurrence and complications rates [12]. Eumycetoma patients characteristically have high drug incompliance and follow-up dropout rates due to the gross patients’ dissatisfaction with the current treatment [12]. Hence, there is a pressing need for novel therapeutic approaches.

Although, plants have been traditionally used as local remedies against various infectious diseases, their activity against mycetoma causative agents have been hardly studied. Only one report appeared to literature which studied the *in vitro* activity of plant extracts such as artemisinin and tea tree oil on *M*. *mycetomatis*. In that study it was demonstrated that that artemisnin was not active against *M*. *mycetomatis*, but tea tree oil did inhibit its growth at concentrations below 0.25% (v/v), which was comparable to other fungal species [18,25]. Tea tree oil is a product not available in the Sudan and hence we tested other local plant species with reported antifungal activities.

Seven locally accessible medicinal plant species were selected and tested for activity against eumycetoma. The study demonstrated variable antifungal activity against *M*. *mycetomatis*, but the plants *Boswellia papyrifera*, *Acacia nubica* and *Nigella sativa* appeared to be the most potent ones. The fact that, extracts of *B*. *papyrifera* showed antifungal activity against *M*. *mycetomatis* is not surprising since the antibacterial and antifungal activities of *B*. *carterii*, *B*. *neglecta*, *B*. *sacra*, *B*. *thurifera*, *B*. *frereana*, *B*. *papyrifera*, *B*. *socrotrana* and *B*. *papyrifera* are widely reported [26][27]. Furthermore, *Boswellia* essential oils were not only able to inhibit the fungal growth, they were also able to inhibit germ tube formation in *C*. *albicans* [26–28] and to prevent Candida *albicans* biofilm formation [26–28–29–30], both important for pathogenicity. Especially the latter feature could be important in mycetoma too. The *Candida* biofilm is composed of *Candida* cells immobilized in matrix, known to consist mainly of carbohydrates and small amounts of protein, hexosamine, phosphorus and uronic acid [29–30]. Likewise, the *M*. *mycetomatis* grain also consists of fungal cells entrapped in a matrix like material called cement [28–29–30]. The composition of the cement differs from that of the *Candida* biofilm in that it is carbohydrate poor, and rich in fat and proteins [29–30], but like the *Candida* biofilm it is highly resistant against antifungal agents. Therefore any compound able to inhibit *Candida* biofilm formation or even to disrupt this biofilm might be able to do the same with the cement material of the *M*. *mycetomatis* grain.

Next to the reported *B*. *papyrifera* antifungal activities, Boswellia species also have known to possess bioactive molecules which are active in inflammatory and malignant diseases and in wound healing [31–32]. The compounds responsible for this anti-inflammatory reaction are thought to be boswellic acids and quercetin [32–34]. From *in vitro* studies and animal models it was learned that, boswellic acids inhibit the synthesis of pro-inflammatory enzymes, 5-lipoxygenase and leukotriene B and thereby prevent bronchoconstriction, chemotaxis, and decrease vascular permeability [32–33]. Furthermore, the anti-inflammatory activity of mixture of boswellic acids produced a 25–46% inhibition of paw oedema in rats and mice [35], which could be important in the mycetoma pathology since large swellings are also present in this disease. Furthermore, since a large inflammation zone is present surrounding the grain (33), any essential oil which also has anti-inflammatory properties will have some effect on the pathogenesis of this disease.

Furthermore, although the *Boswellia papyrifera* extracts in overall appear to be effective and safe to administer [37–38–39–40], identification of the active phytoconstituents is still mandatory. This is important to decrease the probabilities of expected adverse effects and to dramatically increase the prospective extract efficacy when the isolated compounds are used as medication.

The study showed that, the overall the purified stigmatriene MICs were much less than for the crude extracts. This indicates that either stigmatriene is not the only antifungal compound present or that the different components present in the crude extracts might act synergistically. Indeed, in other studies several other compounds were found to be the active ingredient of Boswellia resins [26,27,28]. Based on these observations, more studies are needed on the antifungal mechanism of these extracts before they can be developed as potential new antifungal agents for the treatment of eumycetoma.

In conclusion, the present study showed that, B. *papyrifera*, *A*. *nubica* and *N*. *sativa* have some *in vitro* antifungal activity against *M*. *mycetomatis*, and the most potent were the *B*. *papyrifera* extracts. Although the antifungal activity of the identified compounds in these extracts was only limited, and the antifungal activity of the complete extracts was more promising. Furthermore these plant extracts are also known to have anti-inflammatory activity and stimulate wound-healing and that can be helpful in managing patients with mycetoma.
